# S1PR3 is essential for phosphorylated fingolimod to protect astrocytes against oxygen‐glucose deprivation‐induced neuroinflammation via inhibiting TLR2/4‐NFκB signalling

**DOI:** 10.1111/jcmm.13596

**Published:** 2018-03-13

**Authors:** Yin‐Feng Dong, Ruo‐Bing Guo, Juan Ji, Lu‐Lu Cao, Ling Zhang, Zheng‐Zhen Chen, Ji‐Ye Huang, Jin Wu, Jun Lu, Xiu‐Lan Sun

**Affiliations:** ^1^ Neuroprotective Drug Discovery Key Laboratory of Nanjing Medical University Department of Pharmacology Nanjing Medical University Nanjing Jiangsu China; ^2^ School of Nursing Nanjing University of Chinese Medicine Nanjing Jiangsu China; ^3^ Department of Internal Neurology the Second Affiliated Hospital Nanjing Jiangsu China; ^4^ College of Health Sciences Jiangsu Normal University Xuzhou Jiangsu China

**Keywords:** astrocyte, fingolimod, neuroinflammation, sphingosine‐1‐phosphate receptor 3

## Abstract

Fingolimod (FTY720) is used as an immunosuppressant for multiple sclerosis. Numerous studies indicated its neuroprotective effects in stroke. However, the mechanism remains to be elucidated. This study was intended to investigate the mechanisms of phosphorylated FTY720 (pFTY720), which was the principle active molecule in regulating astrocyte‐mediated inflammatory responses induced by oxygen‐glucose deprivation (OGD). Results demonstrated that pFTY720 could protect astrocytes against OGD‐induced injury and inflammatory responses. It significantly decreased pro‐inflammatory cytokines, including high mobility group box 1 (HMGB1) and tumour necrosis factor‐α (TNF‐α). Further, studies displayed that pFTY720 could prevent up‐regulation of Toll‐like receptor 2 (TLR2), phosphorylation of phosphoinositide 3‐kinase (PI3K) and nuclear translocation of nuclear factor kappa B (NFκB) p65 subunit caused by OGD. Sphingosine‐1‐phosphate receptor 3 (S1PR3) knockdown could reverse the above change. Moreover, administration of TLR2/4 blocker abolished the protective effects of pFTY720. Taken together, this study reveals that pFTY720 depends on S1PR3 to protect astrocytes against OGD‐induced neuroinflammation, due to inhibiting TLR2/4‐PI3K‐NFκB signalling pathway.

## 1 INTRODUCTION

Stroke is currently the leading cause of death and disability worldwide.^1^ Accumulating studies indicated that the pathogenesis of stroke was involved with a series of cascades including energy failure, excitotoxicity, oxidative stress, apoptosis and neuroinflammation.^2^ Among them, neuroinflammation affected the development and progression of stroke, which was primarily mediated by abundant pro‐inflammatory cytokines such as TNF‐α, IL‐1β, IL‐6 and so on in resident glia.^3^ Astrocytes are the most abundant resident glia and play an important role in stroke. During cerebral ischaemia, once astrocytes are activated, they can produce neuroprotective messengers and secrete inflammatory cytokines, and then actively modulates post‐ischaemic inflammation via adaptive and innate mechanisms.^4^

Recently, sphingosine‐1‐phosphate (S1P) is viewed as a new mediator implicated in post‐stroke neuroinflammation, and the mechanisms involved in the S1P's effects have been extensively studied in multiple sclerosis (MS).^5^, ^6^ FTY720, as a synthetic S1P analog, can be phosphorylated by sphingosine kinase. The phosphorylated version pFTY720 could activate S1P receptor 1 (S1PR1) and served as clinical therapy for MS.^7^ Moreover, growing pre‐clinical and clinical studies have proved that FTY720 is effective in treating stroke.^8^, ^9^, ^10^, ^11^ Previous studies suggested that FTY720 protected against stroke not directly acting on neuron but rather on resident and peripheral cells.^8^, ^12^ It has been stated that astrocytes predominantly express S1PR1 and S1PR3, which were essential for their physiological functions.^13^, ^14^, ^15^ Moreover, recent study indicates that FTY720 exerts neuroprotective effect against neuroinflammation mediated by astrocytes.^16^, ^17^ However, the underlying mechanism remains obscure.

Therefore, this study was intended to explore the mechanisms involved in the protective effects of pFTY720 on astrocyte‐mediated inflammatory responses. We found that pre‐treatment with pFTY720 could protect the primary cultured astrocytes against oxygen‐glucose deprivation (OGD) injury and reduce pro‐inflammatory cytokine releases. The results revealed that S1PR3 was essential for the protective effects of pFTY720, due to inactivating Toll‐like receptor 2/4 (TLR2/4)‐nuclear factor kappa B (NFκB) signalling pathway.

## 2 MATERIALS AND METHODS

### 2.1 Drugs and treatments

The phosphorylated version of FTY720 (pFTY720) was purchased from Selleckchem (USA) and dissolved in 90% DMSO and 20 mmol/L HCL and prepared as 10 mmol/L stock solutions. Then, pFTY720 was administrated before OGD 1 hour until to recovery 24 hours at a concentration of 10 nmol/L. The Chinese herb‐derived Sparstolonin B (SsnB) was dissolved in DMSO as 10 mmol/L stock solutions, and diluted in serum‐free media at a final concentration of 10 μmol/L. The cells were pre‐treated 48 hours before treatment with pFTY720.

### 2.2 Primary astrocyte culture

Primary cultured astrocytes were prepared as previously described.^18^ Cortices were aseptically separated from P_0_ SD rats, which were minced and digested with 0.25% trypsin (Gibco, USA) for 10 minutes at 37°C. Then, the dissociated cells were seeded on T75 culture flasks in Dulbecco's modified Eagle's medium (DMEM) (Gibco) containing 10% foetal bovine serum (FBS). The cells were incubated at 37°C carbon dioxide (CO_2_) incubator. The medium was changed every 2‐3 days. After 2 weeks, the flasks were shaken for 1 hour at 37°C to remove other glia. The astrocytes were then detached by trypsinization and plated in 6‐well plates at a density of 1 × 10^6^ cells/mL. All experimental procedures were approved by IACUC (Institutional Animal Care and Use Committee of Nanjing University of Chinese Medicine).

### 2.3 OGD model

OGD and reoxygenation were used as an in vitro model of cerebral ischaemia and reperfusion injury. Briefly, primary astrocytes were washed twice and immersed into glucose‐free DMEM (Gibco) without FBS. Then, they were placed into an anaerobic chamber (Mitsubishi Gas Chemical Company, Japan). After OGD 5 hours, cells were replaced with normal medium containing 10% FBS and returned to the incubator under normoxic condition (37°C, 5% CO_2_) for 24 hours. The control cells were treated similarly except for exposure to OGD.

### 2.4 Cell viability analysis

The MTT assay was used to evaluate the cellular viability as previously described.^19^ Primary astrocytes were seeded onto 96‐well plates at a density of 5 × 10^4^ cells per well. After different time course (3 hour/5 hour/7 hour) of OGD and reoxygenation 24 hours, the MTT (0.5 mg/mL) was dissolved in the cell medium and incubated at 37°C for 4 hours. Then, we used dimethyl sulphoxide (DMSO) to dissolve the MTT‐formazan product. The absorbance was obtained by Dynatech MR5000 plate counter at the test wavelength of 570 nm.

### 2.5 Real‐time PCR

Total RNA (500 ng) was extracted from astrocytes using the TRIzol (Invitrogen) reagent, according to the manufacturer's instruction. The cDNA was synthesized with the SYBR PrimeScript RT‐PCR kit (Takara) and the FastStart Universal SYBR Green Master (ROX) provided by Roche were used to PCR procedure. Crossing threshold (*C*_T_) values were manually set at 2.0 and were normalized to the housekeeping genes β‐actin. Primers for *S1pr1‐S1pr5* of rat are listed in Table 1.

**Table 1:** The primers for PCR amplification

Gene name	Forward (5′ to 3′)	Reverse (5′ to 3′)
GAPDH	GGAGACAACTGGTCCTCCAGTG	ACCTGCCAAGTATGATGACATCA
*S1pr1*	TTCAGCCTCCTTGCTATCGC	AGGATGAGGGAGATGACCCAG
*S1pr2*	GACGCTGGACATGCAGGAG	TACATGGCTGAGTGGAACTT
*S1pr3*	GCCACCCGCCAGTCTTG	GCCAGCTTCCCCACGTAAT
*S1pr4*	CGTTTCCAGCATCCGCAG	CCAGTCCCTTCTCACCTCTCCT
*S1pr5*	CCTATGTGCTCTTCTGCGTGCTG	CGCACCTGACAGTAAATCCTTG
S_1_PR3‐siRNA	CUCCAAAGGUCAAGGAAGATT	UCUUCCUUGACCUUUGGAGTT

### 2.6 Enzyme‐Linked Immunosorbent Assay (ELISA)

The content of inflammatory cytokines including HMGB1 and TNF‐α in the cellular supernatant was evaluated by ELISA kits, which were produced by Cloud‐Clone Corp (USA). All the procedures were in accordance with the manufacturer's instruction.

### 2.7 Western blotting analysis

The cytoplasmic and nuclear protein samples were extracted by the protein extraction kit (Nanjing KeyGen, China), and protein quantity was measured according to the manufacturer's instruction. The supernatants (40 μg protein) were separated by Tris‐glycine SDS‐PAGE, transferred to PVDF membranes and blocked with 10% non‐fat dry milk in TBS containing 0.1% Tween 20 (TBS‐T) for 1 hour at the room temperature. Then, the PVDF membranes were clipped and incubated with primary antibody against TLR2 (Abcam), TLR4 (Novus), S1PR3 (Abcam), p‐PI3K (CST) and total PI3K (CST), GAPDH (Proteintech), H3 (CST), NF‐κB (Bioworld), S1PR1 (Abnova), S1PR2 (Proteintech) and S1PR3 (Abnova) overnight at 4°C. After being washed with TBS‐T for 3 times, they were incubated with corresponding secondary antibody for 1 hour at room temperature. The immunoblots were scanned and determined by Omega 16IC (Ultra‐Lum, USA), and the grey values were calculated by the ImageJ software.

### 2.8 siRNA transfection

Primary astrocytes were transfected with control non‐targeting siRNA or specific S1PR3 siRNAs (listed in Table 1) using lip2000 transfection kit (Invitrogen, USA), according to the manufacturer's instruction. The siRNAs (200 nmol/L, synthesized by Shanghai GenePharma Co., Ltd) were dissolved in the buffer for 5 minutes at room temperature and then added to the siRNA‐buffer solution for 10 minutes. Finally, the siRNA‐reagent complexes were added to the medium and incubated (37°C, 5% CO_2_) for 48 hours.

### 2.9 Statistical analysis

Data were presented as mean ± SEM. Unless stated otherwise, all statistical quantitative assessments were performed in a blinded manner. For 3 or more groups, one‐way analysis of variance (ANOVA) followed by the Student‐Newman‐Keuls tests was used for comparison. Differences were considered significant for *P *<* *.05.

## 3 RESULTS

### 3.1 Pre‐treatment with pFTY720 protects astrocytes against OGD‐induced injury

**Figure 1:**
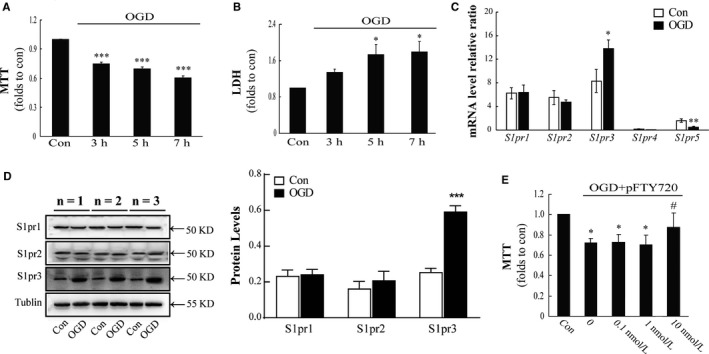
Pre‐treatment with pFTY720 protects astrocytes against oxygen‐glucose deprivation (OGD)‐induced injury. OGD‐induced the changes in cell viability (A) and the releases of LDH in cellular supernatant (B); (C) quantitative analysis of the mRNA levels of S1PR1‐5 expressed in astrocytes; (D) analysis of the protein levels of different S1PR1‐3 expressed in astrocytes; (E) effect of pre‐treatment with pFTY720 on the cell viability. *P <* *.05, ***P <* *.001 vs Con group; #*P *<* *.05, OGD group. Results are shown as mean ± SEM. in every 4 independent experiments. OGD: oxygen‐glucose deprivation; pFTY720: phosphorylated FTY720

In order to uncover the impacts of pFTY720 on OGD‐induced injury, the primary astrocytes were separated from rats and pre‐treated with pFTY720 for 1 hour before exposure to OGD until recovery for 24 hours. The primary cultured astrocytes displayed variation of cell viability and lactic dehydrogenase (LDH) release, subjected to OGD at different time‐points (3, 5 and 7 hours). OGD 3 hours resulted in cell viability decreasing by 25.4% (Figure 1A), but it had no significant effect on LDH release (Figure 1B); when we extended time of OGD to 5 and 7 hours, it decreased by 30% and 40%, respectively (Figure 1A), likewise, there were remarkably increases of LDH in cellular supernatant (Figure 1B). Therefore, exposure to OGD 5 hours was used in subsequent in vitro experiments. To further analyse all the subtypes of S1PRs expressed in astrocyte under normal culturing condition and OGD condition, the mRNA and protein levels of S1PRs were detected by real‐time PCR assay and Western blotting. As shown in Figure 1C,D, the results show that astrocytes expressed high mRNA levels of *S1pr*1, *S1pr2* and *S1pr3*, whereas OGD challenge resulted in significant increases in *S1pr3* and decreases in *S1pr5*. OGD challenge induced similar changes in the protein expression of S1PR1‐3. To observe the roles of pFTY720, we pre‐treated cells with pFTY720 at the different concentrations (0.1, 1 and 10 nmol/L). As displayed in Figure 1E, pre‐treatment with pFTY720 at a final concentration of 10 nmol/L could remarkably attenuate OGD‐induced decreases in cell viability. The results demonstrate that pre‐treatment with pFTY720 prevents astrocytes from OGD‐induced injury.

### 3.2 S1PR3 is essential for the anti‐inflammatory/pro‐inflammatory effect of pFTY720

**Figure 2:**
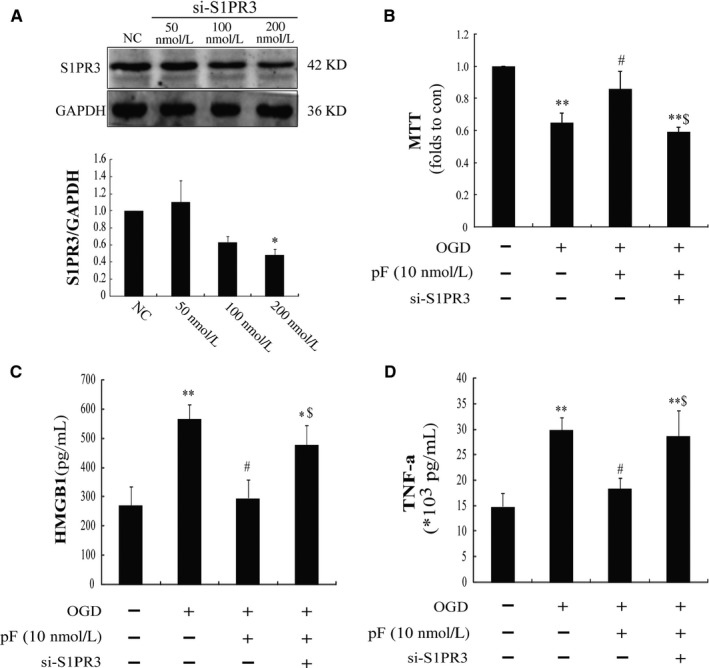
S1PR3 is essential for the anti‐inflammatory effects of pFTY720 in astrocytes. Astrocytes were transfected with s1pr3 siRNA (si‐S1PR3) or negative control (NC) for 48 hr and then treated with oxygen‐glucose deprivation (OGD). Expression of S1PR3 (A), cell viability (B), pro‐inflammatory cytokine levels in the cellular supernatant including HMGB1 (C) and TNF‐α (D) was determined. *P <* *.05, **P <* *.01 vs Con group; #*P *<* *.05 vs OGD group; $*P *<* *.05 vs OGD plus pFTY720 pre‐treatment group. Results are shown as mean ± SEM. in every 6 independent experiments. OGD: oxygen‐glucose deprivation; pFTY720: phosphorylated FTY720

To further define the mechanisms involved in the protective effect of pFTY720, we transfected astrocytes with S1pr3 antisense oligonucleotide to down‐regulate S1PR3. As shown in Figure 2A, the results display that transfection with S1pr3 antisense oligonucleotide (200 nmol/L) could down‐regulate expression of S1PR3 by about 50%. S1PR3 receptor knockdown could abolish the protection of pFTY720 against OGD‐induced damages in astrocytes (Figure 2B). These data suggest that S1PR3 is essential for pFTY720‐mediated protective effects in astrocytes.

Further investigation revealed that there were significant increases in HMGB1 (Figure 2C) and TNF‐α (Figure 2D) after OGD 5 hours in comparison with control groups, whereas pre‐treatment with pFTY720 (10 nmol/L) could observably restrain the increases in HMGB1 and TNF‐α caused by OGD. Moreover, knockdown of S1PR3 could reverse the anti‐inflammatory effects of pFTY720 (Figure 2C,D). These data manifest that pFTY720 can decrease OGD‐induced inflammatory cytokine releases including HMGB1 and TNF‐α, mainly due to S1PR3.

### 3.3 TLR2/4 are involved in pFTY720‐mediated protection against OGD‐induced neuroinflammation in astrocytes

**Figure 3:**
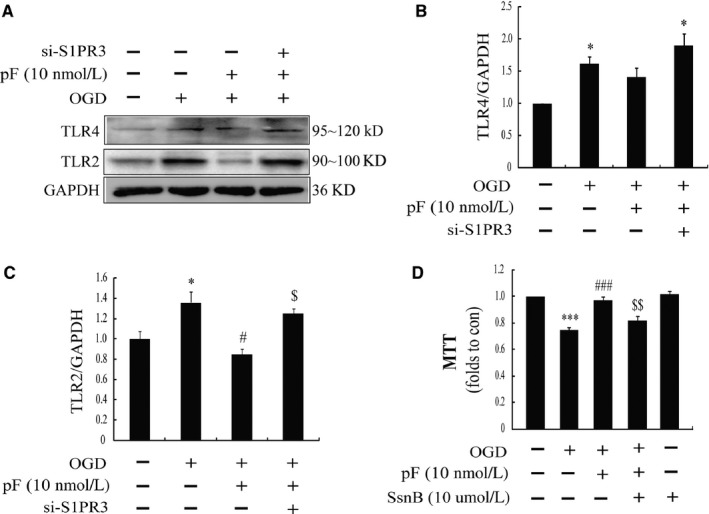
TLR2/4 are pivotal for the protective effects of pFTY720 on oxygen‐glucose deprivation (OGD)‐induced inflammation in astrocytes. (A) Representative immunoblots of TLR2 and TLR4 in astrocytes; the protein expression of TLR4 (B) and TLR2 (C) determined by Western blotting; (D) cell viability determined by MTT assay. *P <* *.05, ***P <* *.001 vs Con group; #*P *<* *.05, ###*P *<* *.001 vs OGD group; $$*P *<* *.01 vs OGD plus pFTY720 pre‐treatment group. Results are shown as mean ± SEM. in every 4 independent experiments. OGD: oxygen‐glucose deprivation; pFTY720: phosphorylated FTY720; SsnB: the Chinese herb‐derived Sparstolonin B (antagonist of TLR2 and TLR4)

TLRs were important receptors expressing in astrocytes, which was critical for neuroinflammation. Among them, TLR2 and TLR4 are the main subtypes. We found that astrocytes expressed both TLR2 and TLR4, and there were significant increases in TLR2 and TLR4 after OGD challenge compared with the control group, whereas pre‐treatment with pFTY720 could down‐regulate the expression of TLR2, but fail to affect OGD‐induced increases in TLR4 (Figure 3A‐C). However, knocking down S1PR3 could reverse pFTY720‐induced regulations on TLR2 expression (Figure 3A‐C). To further clarify the roles of TLR2 and TLR4, the astrocytes were pre‐treated with the Chinese herb‐derived Sparstolonin B (SsnB) (a specific TLR2 and TLR4 inhibitor) before exposure to pFTY720. As illustrated in Figure 3D, the results show that pre‐treatment with SsnB could abolish the protective effects of pFTY720 against OGD‐induced damages in cell viability. These data indicate that TLR2/4 play the critical roles in the neuroprotection of pFTY720.

### 3.4 pFTY720 acts on S1PR3 to regulate the inflammatory cascades via inhibiting PI3K/NFκB signalling pathway

**Figure 4:**
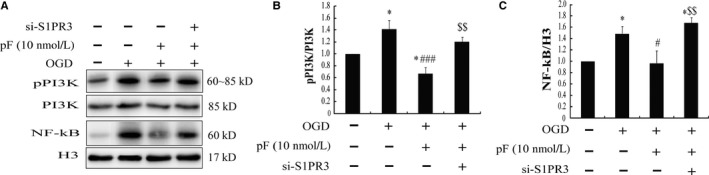
pFTY720 attenuates oxygen‐glucose deprivation (OGD)‐induced inflammation in astrocytes through PI3K/NF‐κB signalling pathway. (A) Representative immunoblots of phosphorylated PI3K and nuclear NF‐κB in astrocytes; the levels of the phosphorylation of PI3K (B) and nuclear NF‐κB (C) determined by Western blotting. *P <* *.05 vs Con group; #*P *<* *.05, ###*P *<* *.001 vs OGD group; $$*P *<* *.01 vs OGD plus pFTY720 pre‐treatment group. Results are shown as mean ± SEM. in every 4 independent experiments. OGD: oxygen‐glucose deprivation; pFTY720: phosphorylated FTY720

Furthermore, we observed the roles of the classical, inflammatory signalling molecules NF‐κB and phosphoinositide 3‐kinase (PI3K) in the anti‐inflammation of pFTY720. The results showed that challenged with OGD significantly induced phosphorylation of PI3K (Figure 4A,B) and also promoted nuclear translocation of NF‐κB (Figure 4A,C). However, pre‐treatment with pFTY720 (10 nmol/L) significantly hampered the phosphorylation of PI3K and activation of nuclear NF‐κB (Figure 4A‐C). Moreover, S1PR3 knockdown could partly reverse the protective effects of pFTY720 (Figure 4A‐C). These results suggest that pFTY720 protects astrocytes against OGD‐induced injury through inhibiting PI3K/NFκB signalling pathway, which is dependent on S1PR3.

## 4 DISCUSSION

The present study provides the evidence that pre‐treatment with pFTY720 can attenuate OGD‐induced injury and inflammatory responses in astrocytes via TLR2/4‐NFκB signalling pathway, which is depended on S1PR3. These findings demonstrate that S1PR3 expressed in astrocytes is pivotal for the protection of pFTY720.

Astrocytes are the most numerous glial cells in the brain, which become reactive after cerebral ischaemia injury. Accumulating evidence supports that neuroinflammation implicated in reactive astrocytes is crucial for brain ischaemic injury.^3^, ^4^ The activated signal of astrocytes is mainly embodied by the morphological changes and increased expression of GFAP. Once activated, astrocytes undergo dramatic morphological changes and secrete inflammatory cytokines.^20^, ^21^, ^22^ OGD could also result in astrocytic activation and production of pro‐inflammatory cytokines, including HMGB1 and TNF‐α. HMGB1 is a potent inducer of post‐ischaemic inflammation,^23^ whose levels correlate with the severity and outcome of ischaemic stroke.^24^ The primary receptors of HMGB1 implicated in brain injury are advanced glycation end products (RAGE), TLR2 and TLR4.^23^, ^25^, ^26^, ^27^ Both TLRs and RAGE are important for mediating astrocytic activation, blockage of TLRs or RAGE signal would suppress activated astrocytes and be beneficial for brain ischaemic injury.^28^, ^29^, ^30^ Our further study found that SsnB, the blocker of TLR2/TLR4, could abolish protection of pFTY720. Previous studies have been proved that both TLR2 and TLR4 were involved in the roles of S1P receptors,^31^, ^32^, ^33^ but the roles of TLR2/4 in OGD‐mediated inflammatory responses in astrocytes need to be clarified.

MAPKs are crucial downstream signal molecules for TLRs and RAGE receptors,^34^, ^35^, ^36^ which primarily activated astrocytes and initiated NF‐κB signalling.^37^ MAPK signalling and NFκB signalling as the important downstream effectors are proposed to be involved in HMGB1‐mediated post‐ischaemic inflammation.^38^ Interruption of the signalling pathways with specific inhibitors or drugs could prevent post‐ischaemic inflammation and is beneficial in treating various diseases, especially for brain injury.^39^, ^40^ Our results demonstrated that pFTY720 pre‐treatment protected against OGD‐induced inflammation in astrocytes, due to regulating PI3K/NFκB signalling pathway.

The anti‐inflammation of pFTY720 was widely approved, which was involved in mobilizing endotheliocyte, recruiting of monocyte and promoting leucocyte.^41^ Most of its therapeutic effects were dependent on S1PR1.^42^, ^43^ In agreement with previous studies,^16^, ^44^ our data showed *S1pr1*,* S1pr2* and *S1pr3* were highly expressed in astrocytes. OGD only induced up‐regulation of *S1pr3*, which indicated S1PR3 might be crucial in OGD‐induced neuroinflammation in astrocytes. It has been extensively studied and confirmed that FTY720 acts on S1PR1, S1PR3 and S1PR5, but has no effects on S1PR2.^45^ Moreover, it has been confirmed that S1PR3 participated in cellular immunity function,^15^, ^46^ and S1PR3 knockout resulted in leucocyte recruitment defects by inhibiting chemotactic functions.^46^ S1PR3 deficiency also attenuated inflammation caused by lung injury.^13^ This study provides a new finding that S1PR3 knockdown abolishes the anti‐inflammatory effect of pFTY720. Our results also prove the pivotal roles of S1PR3 in OGD‐induced inflammatory response in astrocytes.

**Figure 5:**
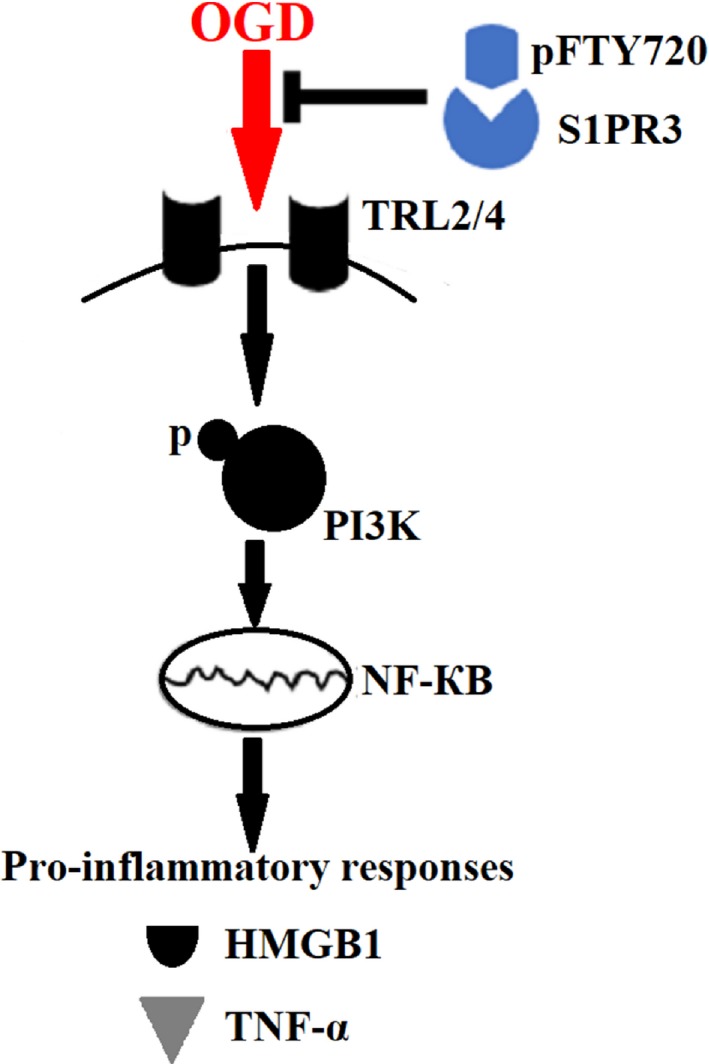
Schematic model. FTY720 depends on S1PR3 to protect astrocytes against oxygen‐glucose deprivation (OGD)‐induced neuroinflammation via inhibiting TLR2/4‐PI3K‐NFκB signalling pathway

In conclusion, this work reveals that pFTY720 depends on S1PR3 to protect astrocytes against neuroinflammation induced by OGD, due to inhibiting TLR2/4‐PI3K‐NF‐κB signalling pathway (Figure 5). Our findings suggest that S1PRs might be a potential therapeutic target for ischaemic stroke.

## CONFLICT OF INTEREST

The authors declare that they have no conflict of interest.
